# Women's experiences with implementation of the physiologic birth program in Iran: a qualitative content analysis study

**DOI:** 10.3389/fgwh.2023.1115365

**Published:** 2023-05-16

**Authors:** Azam Moridi, Parvin Abedi, Mina Iravani, Shahla Khosravi, Narges Alianmoghaddam, Elham Maraghi, Najmieh Saadati

**Affiliations:** ^1^ Department of Midwifery, Nursing and Midwifery School, Ahvaz Jundishapur University of Medical Sciences, Ahvaz, Iran; ^2^ Department of Midwifery, Reproductive Health Promotion Research Center, Ahvaz Jundishapur University of Medical Sciences, Ahvaz, Iran; ^3^ Department of Community Medicine, Faculty Member of Medicine School, Tehran University of Medical Sciences, Tehran, Iran; ^4^ School of Public Health, Massey University, Palmerston North, New Zealand; ^5^ Department of Biostatistics and Epidemiology, Faculty of Public Health, Ahvaz Jundishapur University of Medical Sciences, Ahvaz, Iran; ^6^ Obstetrics and Gynecology, Fertility, Infertility and Perinatology Research Center, Ahvaz Jundishapur University of Medical Sciences, Ahvaz, Iran

**Keywords:** physiologic birth, women experiences, program, Iran, qualitative study

## Abstract

**Introduction:**

Increased rate of caesarean section (CS) without medical indication is a global concern. According to the guidelines of the World Health Organization (WHO), the physiologic birth program is one of the strategies for reducing the rate of unnecessary caesarean sections. The aim of this study is to explain women's experiences with the implementation of the physiologic birth program in Iran.

**Materials and methods:**

This study is a part of a mixed-method study involving 15 targeted semi-structured interviews individually conducted with women attending physiologic birth classes between January 2022 and June 2022. Interviews continued until data saturation was achieved. Data were analyzed using conventional content analysis approach based on the criteria proposed by Graneheim and Lundman, using MAXQDA10 software.

**Results:**

Analysis of the findings of the study led to the emergence of 2 themes, 4 categories, and 10 subcategories. The first theme was the positive experiences of the women (“satisfaction with pregnancy” and “making the childbirth process pleasant”), and the second theme was their negative experiences with physiologic birth (“challenges and limitation of physiologic birth program” and “lack of high-quality obstetric services in the public health system”).

**Conclusion:**

The results of this study showed that childbirth preparation classes reduced women's fear and stress and enhanced their positive attitude toward vaginal delivery by preparing them for childbirth. Also, effective communication with midwives and their support along with efficient implementation of physiologic birth techniques led to successful pain management and satisfaction with the birth process. Policymakers should implement strategies to remove limitations and make this program accessible to all women.

## Introduction

The frequency of caesarean section (CS) is continuously growing around the world, and interventions to reduce unnecessary CS have not been effectively successful (1).

In many cases, cesarean section is performed without medical indication, which can expose pregnant women and their babies to short-term and long-term medical complications. The rate of maternal mortality in CS is four to five times compared with that in normal delivery (2). Mortality rate in natural childbirth is 1.2%, while it is 5.9% in elective cesarean and 18.2% in emergency cesarean per 100,000 live births. In addition, the complications of cesarean section have been reported to be 20%–25% compared to natural childbirth (3). The most common maternal complications generally include infection, bleeding, thromboembolism, and complications of anesthesia, and the most common neonatal complications include scalp laceration, low Apgar score, and cardiopulmonary resuscitation (4).

The rate of CS worldwide has significantly increased in recent decades from less than 7% in 1970 to more than 21% in 2018 and is predicted to increase to 28.5% by 2030 (5). According to the announcement of the Iranian Ministry of Health in 2013, Iran ranked second in the world in the rate of CS (54%) (6). According to the latest report of the Robson classification system, the cesarean rate in Iran was 51.6%, which was up to 90% and higher in private hospitals (7).

According to the guidelines of the World Health Organization (WHO), strategies that can help decrease the rate of unnecessary caesarean sections include promotion of physiologic or non-interventional birth and the use of obstetric care models with an emphasis on childbirth preparation classes aimed at reducing the fear of childbirth and ensuring successful management of labor pain (8, 9). Based on these guidelines, the purposes of this physiologic birth program are to lower the rates of unnecessary medical interventions in the natural process of childbirth, provide personal support for pregnant women by a reliable person, increase the freedom of movement during labor, encourage the use of positions other than lying on the back, and increase immediate mother–infant skin-to-skin contact after delivery and during breastfeeding (8, 9). In the physiologic birth program, a specific emphasis is placed on the control of the fear of childbirth and pain using non-pharmacological pain-reducing methods such as massage therapy, aromatherapy, heat and cold therapy, acupressure, music therapy, reflexology, relaxation, and breathing techniques (10).

The program of preparation classes for childbirth and physiologic childbirth was navigated by the Iranian Ministry of Health in eight hospitals in the country in 2007, and in 2013, this program was implemented in the whole country. The aim of these classes was to promote natural childbirth and reduce instances of cesarean section. Birth preparation classes include eight (90-min) sessions starting from the 20th week of pregnancy, held in some public health centers and private clinics. If there is an accompanying midwife in some hospitals, physiologic birth methods are used for women in labor. The physiologic birth program has been implemented with the general goal of improving maternal and neonatal health and the specific goal of reducing caesarean rates by up to 10% yearly from the base rate in 2013. However, more than 8 years after the implementation of this program, the caesarean rate in Iran is still four to six times higher than the rate recommended by the WHO (11).

The constant rate of vaginal delivery (57%) indicates that the physiologic birth program has not been effective in lowering the rate of caesarean sections and increasing the rate of vaginal delivery based on predetermined goals, even in public hospitals (12). Therefore, it is imperative to investigate the success of this program in the healthcare system (13).

The physiologic birth program is based on the human rights of the women. In the type of childbirth promoted in this program, women's empowerment, freedom of action, and self-control are taken into serious consideration. Their ability to feel self-controlled during the birth process is the basis of a positive childbirth experience (14). Their experiences of childbirth are important criteria for measuring the quality of obstetric care services (15). No qualitative study has hitherto investigated the experiences of women attending the physiologic birth program that is currently implemented in Iran. Investigating the experiences of these women, as recipients of healthcare services, can help identify the implementation of strategies for improving the quality of the physiologic birth program. Therefore, this study was conducted to explore the experiences of Iranian women with the implementation of the physiologic birth program in Iran.

## Materials and methods

### Study design and setting

By using the conventional content analysis approach, this qualitative study was conducted to gain a deep understanding of the mothers' experiences with physiologic birth. This study is a part of a mixed-method study that was started in January 2022 and completed in June 2022 in Ahvaz, Iran. This approach of content analysis, where the researcher allows subcategories and categories to emerge from the text data, adheres to the naturalistic paradigm (16). This paradigm leads to the emergence of profound data which can clarify various dimensions of complex human phenomena (17).

### Data collection and participants

The participants in this study included 15 women. They were eligible to participate in the study if they had given birth, had completed childbirth preparation classes, and had the experience of physiologic birth with an accompanying midwife (up to 6 weeks after delivery). These women were recruited using a purposive sampling method which continued until data saturation was achieved (18), that is, when no new information was revealed with regard to the categories or the relationships between them.

To collect qualitative data, semi-structured individual interviews were conducted with the participants after obtaining their informed consent. The interviews were conducted by the lead researcher, who is a PhD candidate in midwifery (AM). The interviewer was an instructor at the Midwifery Department of the Midwifery and Nursing School with 12 years of teaching and clinical and managerial experience in midwifery. The researcher had completed educational and research courses on qualitative studies and had previously collaborated in qualitative studies as an interviewer.

The time and place of the interviews were agreed upon based on the convenience and choice of the participants without any time limit. They were conducted in private midwifery offices in a separate room where only the interviewer and the participant were present, and the interview continued until deep experiences and detailed answers of participant were obtained. The interviews were recorded using a digital audio recorder. The participants' consent for recording the interview was obtained. Before the outset of the interview, the interviewer attempted to communicate with the participants and created a friendly atmosphere by introducing herself, talking to the participants, and answering their questions. Then, at the beginning of the interview and before its formal commencement, the researcher gave a short verbal explanation about the reasons and objectives of the study and answered any possible question from the participants. This led to the establishment of a good relationship and trust between them. Finally, the participants were assured about their freedom in answering the questions and participating in the research, confidentiality of information, and the possibility of withdrawal from the study at any stage. Given the lead researcher's interest and work experience in the field of physiologic birth, she conducted the interviews and performed data collection and data analysis by leaving aside her previous thoughts and assumptions.

In semi-structured interviews, there are no fixed and predetermined questions, and the questions are formed as the interview unfolds. To start the interview based on the objective of the study, the following general and open question was first asked: “Please tell us about your experiences with the physiologic birth program.” To continue the interview, in-depth and probing questions such as “What do you mean?”, “Why?”, “Please elaborate on that”, and “Could you please give an example so that I understand what you mean?” were asked based on the type of answer given to each question. This was done in order to delve into the depths of their experiences. During the interview, the researcher recorded non-verbal data such as the participants' moods and paralinguistic features like tone of voice, facial expressions, and their posture. Each interview lasted 45–60 min. The interviews continued until data saturation was reached, which was achieved after the 12th interview, but 3 more interviews were also conducted for more assurance. Finally, 15 individual in-depth interviews were conducted with women who had given birth physiologically. No participant was excluded from the study, nor was any interview repeated.

### Data analysis

Conventional content analysis was used for data analysis. The process of data analysis was performed based on the steps suggested by Graneheim and Lundman (19). For this purpose, the researcher transcribed the interview verbatim and performed data analysis at the earliest possible time after conducting the interview, which was generally a few hours after the end of the interview. Then, the researcher read the entire text several times to get a general understanding of the content of the interview. The entire text was considered as the unit of analysis, and smaller parts such as words, phrases, sentences, or paragraphs, which had a meaning or concept related to the research question, were regarded as a meaning unit. Each meaning unit was first converted into condensed meaning units by keeping its original concept, and these units were later coded. The coding process was performed by two authors (AM, PA). The codes were classified into subcategories and categories based on their similarities and differences. Finally, with regard to the hidden meanings of the categories, the themes emerged from the collected data. The data analysis was performed using MAXQDA10 software.

The present study adhered to the four criteria of Lincoln and Guba (20) to enhance the trustworthiness of the data. The credibility of the research data was ensured by the ongoing involvement of the researcher with the subject of the research and spending sufficient time for data collection. Moreover, the content of the categories was reviewed by two participants to ensure the correspondence between the categories obtained and the experiences of the participants. The participants reviewed a short report of the analyzed data for member check to see how well the report reflected their experiences and attitudes. The data were also provided to authors so they could ensure the correspondence between the categories and the statements made by the participants. Dependability was ensured using the opinions of external observers (two midwifery and reproductive health specialists) as well as code–recode method during the analysis. Transferability of the findings was obtained through a detailed description of the context, participants, environment, and conditions. To ensure confirmability, the lead researcher analyzed the data by putting aside her presuppositions and thoughts in all stages of the research including data collection, data analysis, and formation of codes. The opinions of the two midwifery and reproductive health specialists were utilized to reach a consensus on the process of forming the subcategories and categories.

### Ethical considerations

Ethical approval for this study was granted by the Ethics Committee of Ahvaz Jundishapur University of Medical Sciences (**IR.AJUMS.REC.1401.050**). Confidentiality of the collected data was ensured, and written informed consent was obtained from all research participants. In addition, participants were free to decline participation or withdraw at any stage of the research process. All interviews were recorded with the permission of the participants, and all audio files were securely stored in password-protected computers.

## Results

The findings of this study were extracted from the analysis of 15 in-depth individual interviews with women who had the experience of physiologic birth. The characteristics of these women are shown in Table 1. The findings of the study were classified into 2 themes, 4 categories, 10 subcategories, and 90 codes. The main themes were “Positive experiences of physiologic birth program” and “Negative experiences of physiologic birth program” (Table 2).

**Table 1:** Demographic characteristics of the participants (*n* = 15).

Participant	Age (years)	Education	Employment status	Gravidity
1	27	High school	Housewife	2
2	19	High school	Housewife	1
3	21	Bachelor's degree	Housewife	1
4	25	Bachelor's degree	Employee	2
5	29	Bachelor's degree	Employee	1
6	28	Master's degree	Employee	2
7	35	PhD	Employee	1
8	30	Master's degree	Employee	1
9	29	Bachelor's degree	Housewife	1
10	28	High school	Housewife	3
11	26	Bachelor's degree	Housewife	2
12	37	Bachelor's degree	Housewife	2
13	27	High school	Housewife	2
14	31	Bachelor's degree	Employee	1
15	35	High school	Housewife	3

**Table 2:** Themes, main categories, and subcategories extracted from study codes.

Subcategories	Categories	Themes
Constant communication with the midwife	Satisfaction with pregnancy	Positive experiences of physiologic birth
Prenatal preparation		
Positive attitude toward vaginal delivery		
Satisfaction with physiologic birth methods	Making childbirth a pleasant experience	
Receiving professional and family support		
Limited access to free childbirth preparation classes	Challenges and limitations of physiologic birth program	Negative experiences of physiologic birth
No support of insurance organizations for physiologic birth done by midwives		
Limited access to an accompanying midwife		
Low quality of childbirth preparation classes in health centers	Insufficient high-quality obstetric services in the public health system	
Low quality of midwifery services provided by midwives in health centers		

The women's positive experiences of physiologic birth included two categories such as satisfaction with pregnancy and making childbirth a pleasant experience. The category of satisfaction with pregnancy consisted of the three subcategories such as constant communication with the midwife, prenatal preparation, and positive attitude toward vaginal delivery. These are described in more details below.

### Constant communication with the midwife

Based on the experiences of the participants, constant and high-quality communication between the mother and the midwife during pregnancy led to satisfaction with pregnancy. These women believed that receiving prenatal care, attending childbirth preparation classes, and finally giving birth with the help of a selected midwife made their experience of pregnancy and childbirth a positive one. Pregnancy and childbirth create stressful conditions for women, but having a trusted midwife can bring peace to the mother. Continuous communication with a midwife during pregnancy and childbirth is a health need of pregnant women, which can create a positive experience in them by promoting their satisfaction with pregnancy. “During my pregnancy, I always was in contact with my midwife. She treated me very well. You get stressed during pregnancy, but when you have a midwife who can answer your questions whenever you want and when you feel that you always have someone there to help you, you'll feel more comfortable” (P13).

“I insisted a lot that the midwife whom I visited during my pregnancy should come for my delivery. I really got used to her and felt comfortable in her presence. We developed a very good relationship. In my opinion, all mothers need to have a midwife if they want to have a good experience of pregnancy” (P11).

### Prenatal preparation

According to the participants, childbirth preparation classes increased their knowledge about the birth process and reduced their fear and stress, making them more prepared for childbirth. Childbirth preparation classes will help the mother develop her skills in using physiologic birthing methods in the process of labor and delivery. Adoption of non-pharmacological pain reduction methods such as breathing techniques and correcting maternal positions improves maternal and neonatal outcomes.

“I always participated in childbirth preparation classes; they taught me what exercises and movements I had to do so that I can have an easier delivery. This reduced my fear and stress. I used the classes and I was really satisfied” (P2).

“In the classes, they explained different phases of childbirth: what the active phase, the second phase, and the third phase of childbirth are or what we can do to reduce our pain. For example, breathing techniques, movements that make the baby come down more easily, and how to push while preventing any rupture. All of these were explained comprehensively” (P1).

### Positive attitude toward vaginal delivery

The participants in this study highlighted the effect of childbirth preparation classes on choosing vaginal delivery. Pain management techniques for labor and delivery which require no interventions in women will increase their desire to have a natural birth. Therefore, using the principles of physiologic childbirth which is based on controlling pain, reducing the fear of childbirth, and managing the mother's stress during labor and delivery can be effective in reducing the rate of elective cesarean sections in women.

“I'd done a lot of searching on the difference between caesarean section and vaginal delivery and which one to choose. Childbirth preparation classes changed my attitude toward vaginal delivery. I came to know what they do in physiologic birth; how much they help; what the stages of childbirth are; how much breathing help; why everyone used to scream before, but nobody screams now; I learned all of these. Then, I slept on it more and finally decided to choose physiologic birth” (P7).

“Almost all women I knew had experienced caesarean and encouraged me to do the same. But when I took part in these classes, I learned about pain reduction methods and changed my mind. The classes can greatly influence the choice of mothers” (P5).

Another category of positive experiences of physiologic birth was making the childbirth process a pleasant experience which included two subcategories of satisfaction with physiologic birth methods and receiving professional and family support.

### Satisfaction with physiologic birth methods

According to the women participating in this study, physiologic birth techniques such as breathing techniques, posture correction, hydrotherapy, massage, and aromatherapy were effective in the management of pain and the speed of delivery. A good and positive experience of women's previous childbirth affects their willingness to have children and get pregnant again. Women's feeling of empowerment in the process of childbirth and having control over the conditions can reduce the fear of childbirth. The mother feels that she can play an active role in childbirth, and her self-efficacy affects the process of childbirth.

“I had a really good childbirth. The exercises I did in the hospital helped me a lot to continue faster and more easily. Squatting helped me a lot; so did breathing and being in the hot tub. Hot water relaxed the muscles, and that was really great. I felt the baby was coming to the lower part much faster and more easily. It was a good experience which lasted for 2 hours. I didn't think I’d be able to do it so quickly” (P14).

“It was such a good experience that I would like to be pregnant again. I was not so troubled and didn't have too much pain. It had nothing to do with what others said about childbirth and its fears. It was really great. I think it's because of the new methods used recently by midwives; for instance, the oils used for massaging and pressing certain points as well as doing some exercises and other things” (P13).

### Receiving professional and family support

Women with the experience of physiologic birth considered the following as positive and pleasant childbirth experiences: the presence of the accompanying midwife, the creation of conditions for the presence of family members, and the way the midwives treated them in the maternity ward. Psychological and physical support for women from pregnancy to delivery and after delivery creates a different birth experience for women, and women who are supported have a better birth experience. As a professional supporter whose presence comforts the mother, the midwife plays an important role in this respect. Also, the support of the husband or family members helps the mother have a positive experience with her pregnancy and childbirth.

“In the maternity ward, I realized what a blessing I had and thanked God for having an accompanying midwife with me. A woman who came before me was in severe pain, but no one went to that mother at all. The presence of the midwife by my side was a tremendously good experience; if there was no midwife at all I couldn't give birth. She helps you both mentally and physically. First God, then the midwife” (P5).

“As I had selected physiologic birth, I was allowed to have my sister-in-law with me; the midwife provided these conditions for me; I could talk to my husband on the phone and it made me feel better” (P3).

The theme of negative experiences of physiologic birth included two categories such as challenges and limitations of physiologic birth program and lack of high-quality obstetric services in the public healthcare system. The first category included the three subcategories such as limited access to free childbirth preparation classes, no support of insurance organizations for physiologic births attended by midwives, and limitations to access an accompanying midwife.

### Limited access to free childbirth preparation classes

Among the negative experiences of physiologic birth that the participants complained about were the lack of childbirth preparation classes in the health center providing pregnancy care and having to pay for services at private centers. Restrictions on receiving physiologic birth training during pregnancy prevent all pregnant mothers from receiving these services for free. Therefore, these restrictions deprive mothers of these services due to either their lack of knowledge or their inability to pay the fees in a private sector.

“I used to go to the health center for care, but they said they don't have childbirth preparation classes there. Some of the classes were held at another center, but I couldn't participate in those classes because that center wasn't near our home. Otherwise, I would have attended the classes” (P4).

“I didn't know they held some classes in the health center. Are there any classes at all?! If they prepare the ground and hold the classes in public centers such as the one where Ms. ….. refers to, it'll be so good. It will be great for the mothers who can't afford the costs if a midwife comes to the health center during the childbirth” (P13).

### No support of insurance organizations for physiologic births attended by midwives

The participants pointed out the costs they have to pay for private midwives as there are no enough midwives in the public healthcare system for running physiologic birth classes, and insurance organizations provide no financial support for private midwives who run physiologic birth classes and those who attend the delivery. The financial burden of physiologic childbirth creates stress in pregnant women and their husbands. Many women cannot use private physiologic delivery services due to financial limitations, and this can affect not only their mental peace but also the maternal and neonatal outcomes.

“We borrowed some money at the night of delivery as my husband was quite penniless. I really couldn't scrape up the money. It would be great if the government or insurance companies supported us. I was really scared to go to the maternity ward without an accompanying midwife. I asked my husband to borrow some money so that I could go to a hospital with an accompanying midwife. I was worried about what would happen if I couldn't have an accompanying midwife” (P6).

“Although I have supplemental insurance and insisted on using it for my delivery, my midwife said that the insurance doesn't cover the costs of accompanying midwives. Well, mothers can't deal with the costs. I know mothers who would like to have a physiologic birth with an accompanying midwife but can't afford the costs. Less pressure would be on them if the insurance covered at least part of the costs” (P7).

### Limitations of an accompanying midwife

Another factor that contributed to the negative experiences of childbirth among the women participating in this study was the limitation in choosing a hospital with an accompanying midwife. In fact, lack of cooperation with accompanying midwives in all hospitals brought about these negative experiences. The interviewed women stated that they have the right to choose their favorite midwife and hospital. However, any restriction to their right will affect their peace of mind and cause tension in them, their husbands, and their families. Therefore, given the principles of physiologic childbirth and women's desire to choose their known midwives, the corporation of this issue in the health system becomes a necessity.

“Sometimes a woman may want to choose a particular midwife, but that midwife is allowed to go only to a specific hospital. It's not good at all. It would be better if you could choose any midwife at any hospital you want for your childbirth. It's one of the stresses of pregnancy that you don't know exactly which hospital you're going to go with your own midwife” (P8).

“When my pain started, I went to the hospital and told them I want my own midwife to come with me and I called her, but the hospital didn't accept it. They told me that I can only choose from among their accompanying midwives as their gynecologist didn't allow other midwives to come. Although I had supplemental insurance and the hospital was private, they didn't accept it. I talked about it with my midwife, and she asked me to go to Amir al-Momenin Hospital where she was working” (P6).

The second category of negative experiences of the physiologic birth program was the lack of high-quality obstetric services in the public healthcare system. This category included three subcategories such as low quality of childbirth preparation classes held in health centers, lack of high-quality midwifery services provided by midwives in health centers, and lack of education and information for physiologic birth.

### Low quality of childbirth preparation classes in health centers

As asserted by the participants of this study, there was a sharp difference between public and private systems in terms of the quality of childbirth preparation classes. The low quality of childbirth preparation classes in public health centers makes mothers who want to give birth physiologically go to private centers. However, many mothers who cannot afford these centers tend to give up physiologic childbirth.

“I used to go to the health center. The pregnancy classes held at the health center were crowded and had no quality at all. The points taught by midwives in such centers are too brief, as they’re not in a good mood to explain everything in detail; but in private offices, the midwives answer the questions more patiently and explain everything much better. They sent videos and photos and were more responsive. That's why I decided to attend childbirth classes here” (P9).

### Low-quality pregnancy care classes in public health centers

According to the participants, the pregnancy care classes offered by healthcare workers instead of midwives provided almost no advice or information about childbirth preparation and physiologic birth. Pregnancy care should be done by midwives in health centers so that mothers can receive counseling and training related to childbirth preparation classes and physiologic childbirth.

“The health center doesn't have midwives at all. All of them are healthcare workers. They measure weight and blood pressure and record lab tests. If they were midwives and held classes, I would certainly take part in the classes” (P15).

“For my first child, the health center I went to had a midwife who provided all the care I needed. The health center no longer has a midwife now. They used to have a separate room for pregnant women, but it's no longer there. For my current pregnancy, I went there only once and they recorded my tests, but I didn't go there anymore. We just have to go to a private midwife's office” (P9).

## Discussion

This qualitative study was conducted in Iran to explain women's experiences of physiologic birth or the lack thereof. According to the findings of this study, women's experiences were classified into two themes such as (1) positive experiences of physiologic birth and (2) negative experiences of physiologic birth. The positive experiences included two categories such as “satisfaction with pregnancy” and “making the childbirth process a pleasant experience.” Negative experiences consisted of two categories such as “challenges and limitation of physiologic birth program” and “lack of high-quality obstetric services in the public healthcare system.”

According to the results, the significance of communication between the mother and the midwife, as asserted by the participants in this study, creates a positive pregnancy experience and satisfaction with it. Continuous midwifery care is a requirement for any healthcare system seeking improvement of midwifery services, especially by the implementation of midwife-attended physiologic birth. As studies conducted in Iran suggest, continuous care model can improve maternal and neonatal outcomes (21). Based on the evidence on the effectiveness of continuous midwifery care, this model of care is recommended by the WHO during prenatal care. In continuous midwifery care, a midwife or a small group of midwives supports the woman in the prenatal period, during the childbirth, and in the postpartum period (8, 9). The midwifery care model is growing globally in countries such as New Zealand, Australia, the UK, Canada, and Denmark (22, 23) and has long been primary in the Netherlands (24). Although women's access to continuous midwifery care has improved, there is no universal access to it yet. Policymakers and health service providers at the managerial and executive levels should also recognize the significance of developing such a relationship and provide the necessary resources and environment to make it possible (25). The presence of an accompanying midwife in delivery wards as a simple and non-invasive intervention can diminish the anxiety and fear of childbirth in pregnant women and improve their childbirth experiences (26). Many studies around the world have investigated the effect of accompanying midwives on the desire of pregnant women for having physiologic birth and improvement of maternal and neonatal outcomes (27). Given the facts that the continuous midwifery care model is only possible in Iran with privately paid midwives, and that women in the public healthcare system cannot have accompanying midwives, the best solution for the implementation of physiologic birth would be preparing the ground for the presence of accompanying midwives with the support of the government and insurance companies. It seems that the health system in Iran should change the model of receiving care during pregnancy and childbirth in order to be in line with the demands of pregnant women. Considering the high rate of elective caesarean section in Iran, especially in primiparous women, it is necessary to make fundamental changes based on women's demand. The current structure of maternal and neonatal health service providers is not responsive to the needs of the service recipients. Therefore, in order to improve the health of mothers, fundamental changes should be made in the provision of midwifery services, which will move toward the provision of continuous midwifery care models.

The results of the present study revealed that childbirth preparation classes instill a positive attitude in pregnant women to choose vaginal delivery. These results are consistent with those of Camlibel et al. (28) and Andaroon et al. (29). Furthermore, by preparing women for the childbirth process, childbirth preparation classes can reduce mothers' fear and stress. Fear of childbirth and inability to bear the pain of childbirth are obviously the most important reasons why women prefer cesarean section without medical indication (30). Given the increasing rates of caesarean sections around the world, international policies and approaches are being adopted to encourage women to instead choose vaginal delivery (31). Evidence has also shown that participation in childbirth classes reduces childbirth anxiety and creates an acceptance of pain (32). According to the literature and to our study participants, prenatal information and education can lead to effective management of pain during labor and reduced maternal fear and stress, thereby helping the delivery progress through increasing the mother's awareness of the childbirth process. Therefore, the quality of childbirth preparation classes plays a significant role in the successful implementation of the physiologic birth program (33). Although the Iranian Ministry of Health holds childbirth preparation classes in some health centers, the results of the present study showed that not only should the number of these centers be increased but also the quality of these classes needs to be improved, which should be taught by midwives (instead of other healthcare providers) and be assessed by executive managers. The manner in which free childbirth preparation classes are held should be changed so classes could be offered in all public health centers which are more easily accessible to all women. Birth preparation classes should be held by skilled and motivated midwives. Limited access to and poor quality of childbirth preparation classes are one of the obstacles to the successful implementation of the physiologic childbirth program.

According to the recommendations of the WHO, making the birth process a pleasant experience is one of the goals of non-interventional and physiologic birth. Obstetric interventions increase the number of CS, especially emergency ones. Based on the results of the present study, the use of non-pharmacological pain reduction methods leads to positive experiences of childbirth in women. A systematic review showed that although pharmacological methods can reduce pain, they have negative side effects as well (34). Cochrane reviews have also suggested that effective pain relief is not always associated with high maternal satisfaction scores. Although non-pharmacological methods may not reduce the pain of childbirth, supportive communication along with non-pharmacological pain reduction techniques such as relaxation and massage can enhance childbirth satisfaction (35). Women's satisfaction with childbirth is a significant criterion for measuring the quality of childbirth care services. Jafari et al.,(15) found that the following factors are associated with the mother's satisfaction with childbirth: improving the physical structure and the decoration of the delivery room, non-pharmacological pain relief, mother's participation in the delivery process, and psychological support. Our results are in line with the above-mentioned studies. What lies at the heart of the principles of physiologic childbirth is to make women's pregnancy and childbirth experiences pleasant, which can be effective both in choosing the mode of delivery and in the progress and management of labor and delivery. The sense of empowerment and self-efficacy that the mother gets creates a positive experience of pregnancy and childbirth. Therefore, it definitely affects the subsequent desire to the next pregnancy and, of course, the choice of natural childbirth.

According to the results of the present study, some of our study participants had positive experiences of physiologic childbirth. However, those who did not have access to childbirth preparation classes taught by midwives or could not have midwife-accompanied births complained about the negative birth experiences caused by the socio-structural limitations as noted above. In line with these results, Songül Aktaş et al. (36) found that inadequate communication of childbearing women with healthcare professionals and ineffective reproductive healthcare policies lead to negative childbirth experiences (36). Low-quality midwifery services or the lack of midwives in the Iranian public healthcare system limit communication between pregnant mothers and midwives. Betran et al. (1) also found that midwife-led care is correlated with higher rates of physiologic birth, safer outcomes, lower healthcare costs, and positive maternal experiences in high-income countries. Thus, such approaches should be evaluated and implemented in middle- and low-income countries as well. The low quality of midwifery services in the Iranian public healthcare system makes mothers prefer private midwifery care when they can afford these. Moreover, lack of support of insurance organizations in providing midwifery services in the private sector leads some pregnant women to develop negative feelings about their childbirth experiences. Khosravi et al. introduced two strategies for improving the quality of midwifery care and midwifery-oriented care in Iran: the support of insurance companies and defining the position of midwives in the referral system (37). Accordingly, there is a need to change the current delivery care model provided by non-midwives in health centers and move toward quality midwifery care with a focus on childbirth preparation classes and physiologic birth. In Iran, physiologic birth is a new and transformative psychological experience that creates a sense of empowerment in women. The benefits of this process can be maximized through providing physical, emotional, and social support for women, increasing their trust in their ability to give birth, and not receiving interventions that disrupt the physiology of labor unless they are truly medically indicated (38). Healthcare providers who are responsible for providing care for pregnant mothers in a public healthcare facility must have the necessary awareness and knowledge of and trust in physiologic childbirth and its positive psychological effects. This will help them provide women with the necessary training and counseling to attend childbirth preparation classes and to choose to have physiologic births, which constitute important parts of the field of the midwifery profession. The impact of the role of midwives in the process of pregnancy and childbirth should be taken into serious consideration by health policymakers.

Many studies have confirmed the effects of education on the promotion of physiologic birth. Training classes during pregnancy provide good opportunities for raising the awareness of women and their families and changing their attitudes toward childbirth. Based on these studies, improving women's knowledge and information through promoting their view of childbirth as a natural process and trust in the body's inherent ability to give birth can increase their self-confidence to choose physiologic birth (39, 40). As such, childbirth preparation classes taught by midwives are required as an essential and standard part of prenatal care in healthcare centers. Physiologic delivery program aimed at reducing the number of cesarean sections should be considered an important health priority by health policymakers. This is because natural childbirth in conventional ways that are currently implemented in Iranian public health centers may not increase the desire of women to choose natural childbirth. Therefore, there should be a change in women's attitude toward childbirth, with the focus on making the process of pregnancy and childbirth pleasant.

### Strengths and limitations of the study

This is the first scholarly attempt to evaluate women's experiences with the implementation of the physiologic birth program in Iran. However, this study is limited in that it was conducted only in private midwifery offices because physiologic birth in Iran is only supported by private midwives.

## Conclusion

The results of this study showed that childbirth preparation classes reduced women's fear and stress and enhanced their positive attitudes toward normal vaginal delivery by preparing them for childbirth. In addition, communication and support of midwives and implementation of physiologic birth techniques were found to lead to effective pain management and satisfaction with the birth process. Health policymakers should implement strategies to remove limitations and make this physiologic birth program accessible to all women in Iran. The positive and negative experiences of women with physiologic birth should be taken into serious consideration by health policymakers in order to improve maternal and neonatal health and promote physiologic birth. Strategies such as implementation of obstetric care models, support for accompanying midwives, quantitative and qualitative improvement of childbirth preparation classes, and provision of quality obstetric services in healthcare centers can be used for improving the quality of the physiologic birth program.

## Data Availability

The raw data supporting the conclusions of this article will be made available by the authors, without undue reservation.
